# Quality assessment of pre- and postnatal nutrition and exercise mobile applications in the United States and China

**DOI:** 10.3389/fnut.2022.942331

**Published:** 2023-01-09

**Authors:** Hongli Yu, Juan He, Keqiang Li, Wen Qi, Jiahui Lin, Anna Szumilewicz

**Affiliations:** ^1^Department of Sport, Gdansk University of Physical Education and Sport, Gdańsk, Poland; ^2^Jiuling Primary School, Mianyang, Sichuan, China; ^3^Football Academy, Beijing Sport University, Beijing, China

**Keywords:** maternity applications, models, quality, nutrition, physical activity

## Abstract

**Background:**

Mobile applications (apps) are becoming increasingly prevalent as tools for improving maternal health behaviors. However, the recently updated content and quality of these apps remain unknown. This research investigated the fundamental characteristics, functional modules, and overall quality of maternal apps available in the United States and China to reveal critical nutrition and physical activity gaps.

**Methods:**

A systematic search was performed in Android and iOS app stores (China and the United States). Apps were eligible if they targeted pregnant or postpartum women, focused on nutrition or physical activity, and had interfaces in English or Chinese. The basic characteristics, functional modules, and overall quality of the apps were evaluated, and differences between apps available in China or the United States were determined using analysis of variance and chi-square tests. Pearson correlations were utilized to investigate links between objective quality and user rating.

**Results:**

A total of 65 maternity-related nutrition and physical activity apps (34 from China and 31 from the United States) were eligible. Among them, 68% (21/31) of US apps and 56% (19/34) of Chinese apps did not provide supporting evidence for their content. A greater number of Chinese apps provided app-based general education modules, namely food nutrition knowledge (*n* = 0, 0% in the United States vs. *n* = 30, 88.2% in China). Meanwhile, a greater number of US apps provided exercise modules, namely pregnancy yoga (*n* = 21, 67.7% in the United States vs. *n* = 2, 5.9% in China). The overall app quality rating in the United States was lower than it was in China (mean: 3.5, SD: 0.6 in China vs. mean: 3.4, SD: 0.7 in the United States). There was no relationship between the overall app quality rating and the user rating in either country (rho = 0.11 in China and rho = –0.13 in the United States).

**Conclusion:**

The characteristics and functional modules of in-store apps for maternal nutrition and physical activity differed between the United States and China. Both countries’ apps, especially Chinese apps, lacked evidence-based information, and there was no correlation between app quality and user rating. The results therefore suggest that user ratings cannot be used as an objective indicator of app quality and that it is necessary to improve the empirical basis and credibility of apps in both countries.

## 1. Introduction

Women’s health is at its most susceptible during the pregnancy to postpartum (PtP) stages (1, 2). Unhealthy eating habits and physical inactivity are prominent health risk factors throughout the PtP stages. They may cause both short- and long-term health issues for women (3, 4), such as the need for obstetric intervention (e.g., cesarean section and instrumental delivery) (5), a greater likelihood of fetal macrosomia (i.e., a large-for-gestational-age baby) (6), a greater risk of pregnancy-related complications (e.g., gestational diabetes, hypertension, pre-eclampsia) (7), and poor cardiovascular health (8).

Some studies have found that obtaining the appropriate amount of nutrition and physical activity (N&PA) throughout the PtP period is crucial for the health of both mother and newborn as well as for the long-term health of both mother and child (9–12). Consequently, the amount of N&PA required at each phase of PtP must be meticulously planned and managed to reduce the incidence of complications. However, recent researches in Canada and Australia suggested that fewer than 50% of women receive suitable guidance from professionals about healthy N&PA during the PtP period (13, 14). This might be due to healthcare being too expensive or inaccessible in low-income regions (15) or to the coronavirus pandemic (COVID-19) and associated isolation periods (16, 17).

The tremendous surge in popularity of mobile health (mHealth) and electronic health (eHealth) has changed the conventional model of healthcare services in recent years (18). More people consult health and lifestyle information *via* mobile applications (apps), which have the potential to significantly improve current therapy and minimize reliance on professional team health services (19, 20). For instance, 81% of Chinese (21), 75% of Australian (22), and 93% of Swiss (23) prenatal and postnatal women reported using at least one prenatal and postnatal app, with more than a quarter reporting that learning about N&PA was the primary reason for doing so. Furthermore, health-related apps have contributed to safe social distancing and lower infection rates during the COVID-19 pandemic and its associated isolation periods (24). As these apps have become essential digital resources for accessing N&PA guidance throughout the PtP period, increasing amounts of research have been devoted to assessing their quality and efficacy to ensure that women receive appropriate guidance. Meanwhile, the Mobile Application Rating Scale (MARS), a reliable, multi-dimensional, simple, and objective tool, was thus developed to classify and evaluate the quality of mobile health apps. It may be used to provide a checklist for designing and developing new high-quality health apps (25).

Evidence from systematic reviews and randomized controlled trials (RCTs) suggests that pregnancy and postpartum apps are generally effective in promoting maternal and newborn physical health (e.g., weight management, postpartum recovery, and infant care) (7, 12, 26). In addition, their cost-effectiveness and convenience have been beneficial during the COVID-19 pandemic (27). Nevertheless, the apps quality (esthetics, functionality, information, and engagement) varies significantly. Many lack supporting RCTs, rigorous evaluation procedures, or published protocols and generally show an absence of quality assessment (17, 28). However, new users still choose to download such apps, and research has shown that user feedback (subjective quality) is more influential in convincing new users to download an app than RCTs and scientific evidence (29). The correlation between user feedback and app quality is particularly important for new users to choose high-quality apps. Unfortunately, the correlation between user feedback and app quality has not been thoroughly investigated based on previous studies. Furthermore, most research on these apps was conducted before 2020, and previous reviews of N&PA apps have tended to focus on pregnant women while disregarding the pre-conception and the postpartum period (21, 30, 31). Finally, some reviews evaluating the information and quality of apps restricted their search to those available in their respective country’s app store (30, 32–34). Consequently, many frequently used apps may have been excluded, thereby limiting the conclusions that can be drawn from the results.

Previous research on the effect of N&PA apps during the PtP period revealed that the influence of mobile apps on prenatal and postnatal women’s lifestyle, health, and decision-making is significant. However, data from RCTs on this subject are still insufficient. Noteworthy gaps in the current research were also exposed. First, the quality and efficacy of recently updated PtP N&PA apps are unknown, which may hinder health improvement, as misinformation about N&PA could lead to inappropriate decision making and increase the risk of adverse health outcomes during the perinatal period (35). Second, the link between user rating and app quality rating has been ignored; as a quick way to judge the quality of an app, user ratings help drive new users to download them (36). Third, commonly used apps may have been missed.

To address the above-mentioned gaps, the aims of this study were (1) to describe and analyze the characteristics and functional modules of PtP N&PA apps in the United States and China, two of the largest app markets; (2) to comprehensively evaluate the overall quality of PtP N&PA mobile apps; and (3) to investigate the connection between app quality rating and user rating. We hypothesized that (1) the recently updated information offered by apps would be consistent with N&PA guidelines in PtP period; (2) PtP N&PA apps in the United States and China would have comparable characteristics and functional modules; and (3) there would be a link between app quality and user rating.

## 2. Materials and methods

### 2.1. Search strategy

A systematic search was performed in Tencent My App (China), Huawei App Market (China), 360 Mobile Assistant (China), Apple App Store (China and the United States), and Google Play (United States) from January 3 to February 27, 2022. Apps were searched using the following keywords: “diet,” “food,” “nutrition,” “supplement,” “food intake,” “nourishment,” “healthy eating,” “food safety,” “snack,” “soft drink,” “carbonated beverages,” “vegetarian,” “nutrition recommendations,” “nutrition guidelines,” “vegan,” “fruit,” “vegetable,” “physical activity,” “physical fitness,” “exercise,” and “sport” in both the Chinese and English languages. To narrow the results, each search word was combined with “pregnancy,” “pregnant,” and “postpartum.”

### 2.2. Selection process

The apps included in this research were defined as those that provided any dietary or physical activity content related to the pre-conception, pregnancy, or postpartum periods. In the preliminary search list, only apps with >1 million installations were included. According to user feedback, consumers are less inclined to pick and install mobile apps with fewer downloads than this (29). The following apps were excluded: (1) those without a rating; (2) those without a Chinese- or English-language interface; (3) those without detailed function description and content introduction in the app store; (4) unrelated and duplicate apps; (5) paid apps with no trial period; (6) those with no updated version since December 31, 2019; and (7) those with <1 million installations.

### 2.3. Data extraction

Two independent researchers selected the apps for study inclusion based on the eligibility criteria. Apps that fulfilled the inclusion criteria were downloaded on either an iPhone or an Android device. The researchers collected the following information from each of the eligible apps: developer, name, app category, latest update date, in-app purchase, target audience, app store, safety statement, privacy policy, evidence-based information (i.e., RCTs or published scientific literature information of app were provided in the app introduction or in the content of the app or the app official website), languages, average user rating, and app functionalities (Note: the preview or function description in the app store would be a reference if the developer had not set up the functional module partition). To prevent data loss, each researcher compiled the above information in a standard spreadsheet. Inconsistencies in extracted data between the researchers were addressed through discussion or consultation with a third researcher.

### 2.4. Content analyses

The Mobile Application Rating Scale (MARS) was used to assess app quality across four dimensions: engagement (five items), functionality (four items), esthetics (three items), and information quality (seven items) (37). The MARS subjective quality subscale is based on the user rating as a subjective evaluation of the user experience. All MARS elements were rated using a five-point Likert scale (1 = inadequate to 5 = excellent) (37). Overall app quality rating was measured by averaging the four dimensions, with each dimension’s mean score used to calculate the overall mean score (37). Apps with a MARS score of three or more but less than four were considered to be of satisfactory quality, while those with scores of four or more were considered to be of high quality (25). Six MARS-trained researchers were assigned an equal number of apps to independently assess app quality using MARS and record the functionalities. All researchers registered each included app to record functionalities, assess and rate its functionality, engagement, esthetics, and information quality and to ensure that each app element was evaluated. A simulated data set of the beginning of the preceding menstrual cycle and an expected delivery date were inputted where relevant to correctly evaluate the potential of the apps. Two researchers acted in the first trimester, two in the second trimester, and two in the third trimester to fully review an app’s functionality throughout pregnancy. All the researchers gathered and evaluated data from the trying to conceive (TTC) and postpartum periods. Each researcher used the same spreadsheet to record the primary and secondary functionalities of each module after signing into the app. Between February 28 and March 20, 2022, the researchers gathered and evaluated data on the apps’ basic characteristics, functional modules, and quality. Considering the influence of researcher’s subjective perception, the reliability of the data collected by the six researchers was examined using the Fleiss Kappa value, with a Kappa value <0.2 indicates poor consistency, 0.2∼0.4 indicates average consistency, 0.4∼0.6 indicates moderate consistency, 0.6∼0.8 indicates strong consistency, and 0.8∼1 indicates very strong consistency (38). The researchers discussed disparities and uncertainties among the in-app ratings to come to an agreement on the final MARS scores.

### 2.5. Statistical analyses

Origin (2021) (Northampton, MA, USA) was used to calculate the frequency of basic characteristics and functional modules, and Statistica (version 14.0) was used to conduct Kolmogorov-Smirnov test, an analysis of variance (ANOVA), and chi-square test. The Statistical Product and Service Solutions (SPSS) version 26.0 was used to calculate the Fleiss Kappa value for the consistence of data collected by six researchers. ANOVA was used to examine the user rating difference between the Chinese and US apps, with a significant threshold of 0.05. A chi-square test was used to examine the characteristics and functionality differences between the Chinese and US apps with a significance threshold of 0.05. The associations between overall app quality rating and user rating were examined using Pearson correlations with significance thresholds set at *p* < 0.05.

## 3. Results

### 3.1. App selection

**FIGURE 1:**
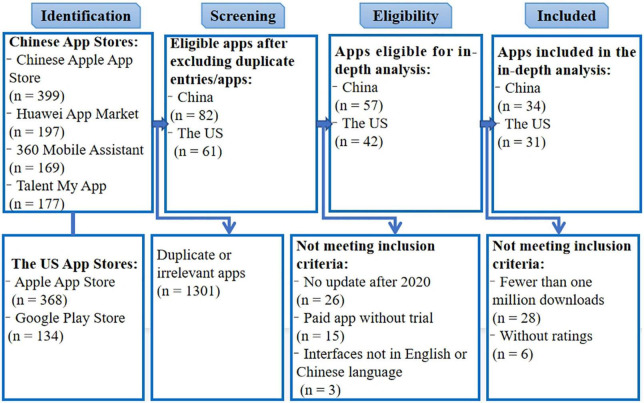
Flowchart of the selection process for apps included in the review.

Based on the search strategy, 1,444 apps were identified and screened in Tencent My App, Huawei App Market, 360 Mobile Assistant, Apple App Store, and Google Play; 1,379 were excluded, with one of the Chinese apps excluded through discussion with a third researcher since the two researchers had different viewpoints after comparing two Chinese apps with different names and developers but the same content and functions. After further exclusions, a total of 65 apps (31 from the United States and 34 from China) were included (Figure 1).

### 3.2. Basic characteristics

Table 1 summarizes the basic characteristics of all the included apps. The Kolmogorov-Smirnov test results showed that all the analysis items were not significant (*p* > 0.05), indicating a normal distribution. In the United States, all the apps were categorized by the app stores: 74.2% (23/31) were categorized as “health and fitness,” while just 9.7% (3/31) were labeled as “medical.” An in-app update comparison found no substantial difference between the US and Chinese apps (*p* > 0.05). Regarding in-app purchase, the US app stores had a higher percentage of in-app purchases than did those in China: 71% (22/31) in the United States vs. 32.3% (11/34) in China; *p* < 0.01. The average user rating of US apps was significantly lower than that of Chinese apps: mean = 3.4 in the United States vs. mean = 3.8 in China; *p* < 0.05. In China, 8.8% (3/34) of mobile apps were categorized as “medical” and 55.9% (19/34) as “health and fitness,” while 2.9% (1/35) were unclassified. A total of 47.1% (16/34) of Chinese mobile apps lacked explicit privacy policy and safety statements, while 80.7% (25/31) of US apps supplied clear safety statements (*p* < 0.05), and 87.1% (27/31) provided a privacy policy (*p* < 0.01). Further, a greater proportion of Chinese apps than US apps targeted breastfeeding mothers and babies [lactating women: 73.5% (25/34) in China vs. 3.2% (1/31) in the US; baby: 79.4% (27/34) in China vs. 3.2% (1/31) in the US; *p* < 0.01]. Multiple language support was present in just 8.8% (3/34) of Chinese apps but 25.8% (8/31) of US apps (*p* < 0.01).

**TABLE 1:** Characteristics of the 65 nutrition and physical activity apps for prenatal through postpartum evaluated in a United States–China comparison.

Category	China (*n* = 34)	United States (*n* = 31)	χ ^2^/*F*	*P*
**Specifications, *n* (%)**				
Food and drink	9 (26.47)	5 (16.13)	4.395	*p* > 0.05^2^
Health and fitness	19 (55.88)	23 (74.19)		
Education	1 (2.94)	0		
Life	1 (2.94)	0		
Medical	3 (8.82)	3 (9.68)		
NA^4^	1 (2.94)	0		
**In-app purchase, *n* (%)**				
With	11 (32.35)	22 (70.97)	9.674	*p* < 0.01**^2^
Without	23 (67.65)	9 (29.03)		
**Target users (app description accompanied by a clear statement), *n* (%)**				
Women TTC^1^	3 (8.82)	9 (29.03)	36.744	*p* < 0.01**^2^
Men TTC	2 (5.88)	0		
Baby	27 (79.41)	1 (3.23)		
Lactating women	25 (73.53)	1 (3.23)		
Pregnant women	26 (76.47)	13 (41.94)		
Postpartum women	28 (82.35)	24 (77.42)		
**Safety statement, *n* (%)**				
With	18 (52.94)	25 (80.65)	5.558	*p* < 0.05*^2^
Without	16 (47.06)	6 (19.35)		
**Privacy policy, *n* (%)**				
With	18 (52.94)	27 (87.10)	8.880	*p* < 0.01**^2^
Without	16 (47.06)	4 (12.90)		
**Evidence-based information, *n* (%)**				
With	15 (44.12)	10 (32.26)	0.964	*p* > 0.05*^2^*
Without	19 (55.88)	21 (67.74)		
**Operating system, *n* (%)**				
iOS and Android iOS	12 (35.29) 10 (29.41)	8 (25.81) 13 (41.94)	1.237	*p* > 0.05*^2^*
Android	12 (35.29)	10 (32.26)		
**Year of the most recent update, *n* (%)**				
2020	12 (35.29)	5 (16.13)	0.039	*p* > 0.05^2^
2021	16 (47.07)	20 (64.52)		
2022	6 (17.64)	6 (19.35)		
**Language, *n* (%)**				
Chinese	31 (91.18)	0	37.670	*p* < 0.01**^2^
English	0	23 (74.19)		
Multiple languages offered	3 (8.82)	8 (25.81)		
Mean user rating (stars/5)	3.75 ± 0.25	3.39 ± 0.77	6.414	*p* < 0.05*^3^

^1^Trying to conceive.

^2^Chi-square test.

^3^Analysis of variance.

^4^Not available.

**Extremely significant difference at *p* < 0.01.

*Significant difference at *p* < 0.05.

### 3.3. Functional modules

**FIGURE 2:**
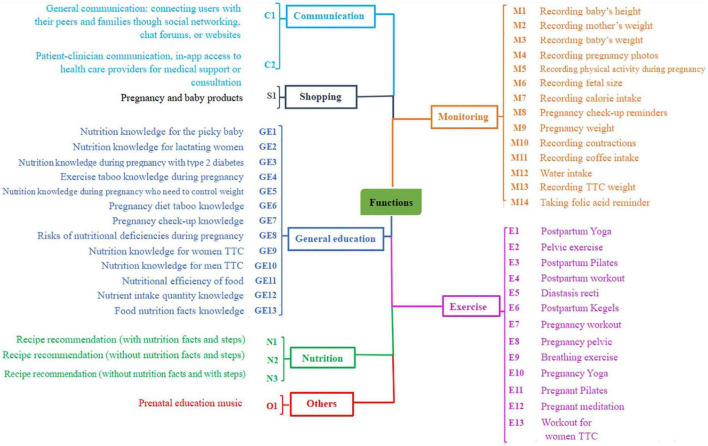
The mind map depicts the primary and secondary models available in the United States and Chinese apps. TTC, trying to conceive.

**FIGURE 3:**
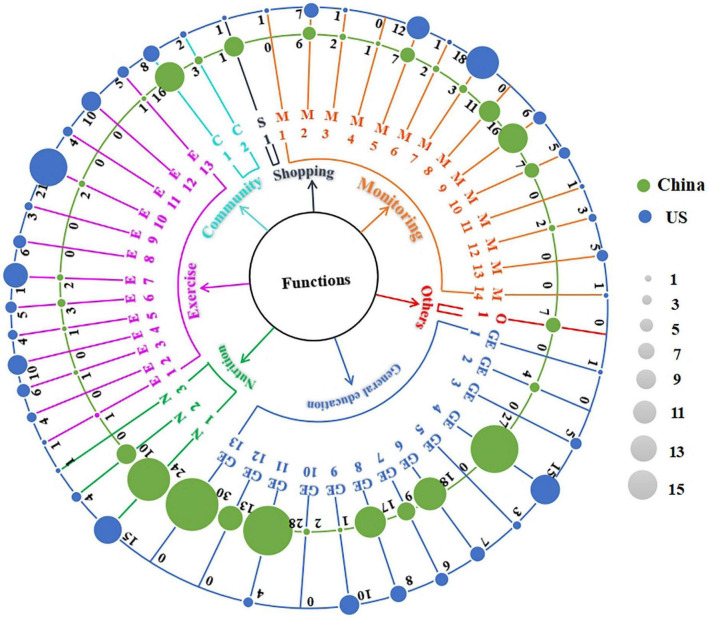
The bubble chart compares the secondary models of 34 Chinese and 31 United States-based mobile apps for prenatal to postnatal nutrition and physical activity. Abbreviations used in this figure are detailed in Figure 2.

The primary and secondary functionalities of each module supplied by the US and Chinese apps are depicted in Figure 2. Both US and Chinese PtP N&PA apps included seven common modules (general education, exercise, monitoring, shopping, community, nutrition, and others) and five categories of targeted users (men TTC, women TTC, infant, pre- and postpartum women). The comparison of functional modules supplied by the US and Chinese apps is depicted in a bubble chart (Figure 3). Analysis items conform to a normal distribution (*p* > 0.05). Pregnancy yoga was the most prevalent course of exercise module among the 31 US apps (*n* = 21, 67.7%), followed by calorie tracking in monitoring module (*n* = 18, 58.1%) and pregnancy-related exercise taboo knowledge in general education module (*n* = 16, 51.6%). Food nutrition knowledge in general education module was the most prevalent content among the 34 Chinese apps (*n* = 30, 88.2%), followed by nutritional effectiveness of food (*n* = 28, 82.4%) and pregnancy-related exercise taboo knowledge (*n* = 27, 79.4%). None of the 34 Chinese apps contained the following components: recording the baby’s height, documenting coffee consumption, recording TTC weight, setting a folic acid reminder, nutrition knowledge during pregnancy with type 2 diabetes, nutrition knowledge for weight management during pregnancy, pelvic exercises during pregnancy, breathing exercises during pregnancy, or meditation during pregnancy. The following components were not available in any of the 31 US apps: pregnancy image recording, pregnancy check-up reminders, nutrition knowledge for lactating women, TTC nutrition knowledge for males, nutrient intake amount knowledge, food nutrition knowledge, or baby music.

The ANOVA results indicated the following significant disparities in modules between the US and Chinese apps: (1) general education: pregnancy diet taboo knowledge (*n* = 7, 22.6% in the United States vs. *n* = 18, 52.9% in China), nutrition knowledge for women TTC (*n* = 10, 32.3% in the United States vs. *n* = 1, 2.9% in China), nutrition knowledge during pregnancy with type 2 diabetes (*n* = 5, 16.1% in the United States vs. *n* = 0, 0% in China), food nutrition knowledge (*n* = 0, 0% in the United States vs. *n* = 30, 88.2% in China); (2) nutrition: recipe recommendations (with nutrition facts and steps) (*n* = 15, 48.4% in the United States vs. *n* = 24, 70.6% in China); (3) exercise: meditation during pregnancy (*n* = 10, 32.3% in the United States vs. *n* = 0, 0% in China), pregnancy yoga (*n* = 21, 67.7% in the United States vs. *n* = 2, 5.9% in China), and pregnancy workouts (*n* = 13, 41.9% in the United States vs. *n* = 0, 0% in China) (*p* < 0.01) (Figure 2).

### 3.4. App quality

**FIGURE 4:**
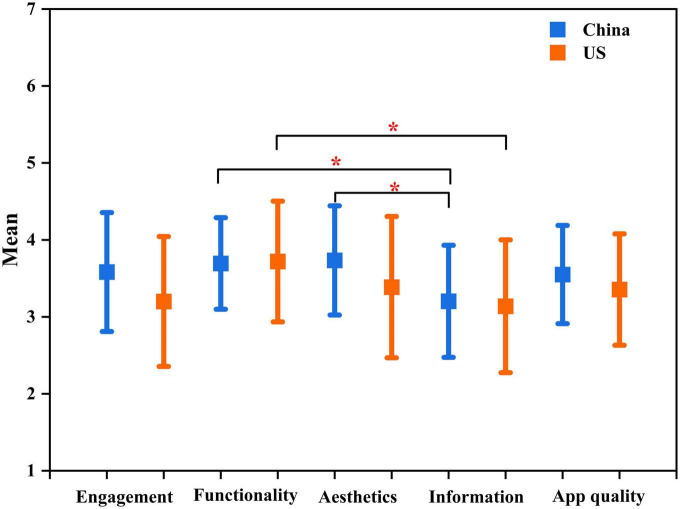
Mean difference in the engagement, functionality, esthetics, information, and overall app quality scores between included apps from the United States and China. *p < 0.05.

Inter-rater reliability for the four dimensions of app quality was satisfactory (Kappa value = 0.79). The overall MARS ratings for app quality ranged from 2.7 to 4.7 (mean: 3.5, SD: 0.6) in China vs. 1.8 to 4.5 (mean: 3.4, SD: 0.7) in the United States, with the majority of apps (21/31, 67.7% in the United States vs. 29/34, 85.3% in China) scoring at or above three (*p* > 0.05). The engagement score ranged from 2.8 to 5 (median 3.6, SD: 0.8) in China vs. 1.6 to 4.2 (mean 3.2, SD: 0.8) in the United States (*p* > 0.05). The functionality score ranged from 2.8 to 4.8 (mean 3.7, SD: 0.6) in China vs. 1.8 to 4.8 (mean 3.7, SD: 0.8) in the United States (*p* > 0.05). The esthetics score ranged from 2.3 to 5 (mean 3.7, SD: 0.7) in China vs. 1.7 to 5 (mean 3.4, SD: 0.9) in the United States, and the information score ranged from 2.2 to 4.4 (mean: 3.2 SD: 0.7) in China vs. 1.7 to 4.5 (mean 3.1, SD:0.9) in the United States (*p* > 0.05). The Kolmogorov-Smirnov test results showed that overall app quality, engagement, functionality, esthetics, and information scores were not significant (*p* > 0.05), indicating a normal distribution. The functionality, esthetics, and information scores varied significantly in the Chinese apps, and a significant difference between functionality and information scores was identified in the US apps (*p* < 0.05) (Figure 4). Supplementary Table 1 presents the mean MARS scores for all 65 assessed apps from the United States and China.

### 3.5. Relationship between app quality and user rating

**FIGURE 5:**
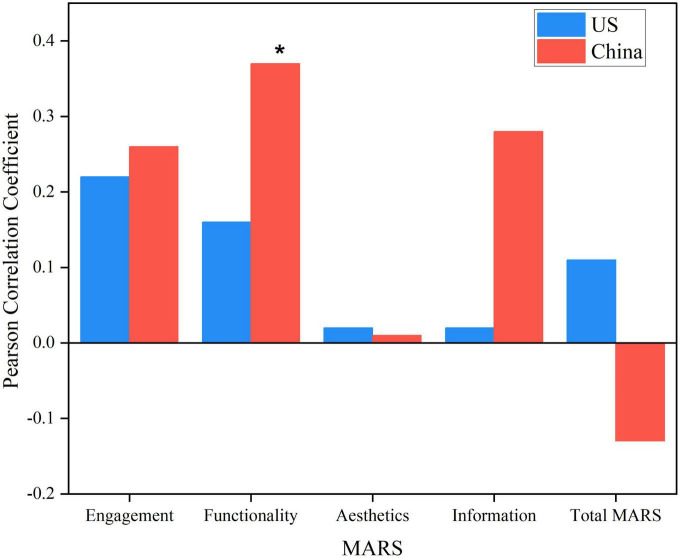
The bar chart illustrates the Pearson coefficients between Chinese and United States mobile app quality rating, four dimensions rating of app quality, and user rating. The Pearson coefficients’ maximum positive and negative limits are indicated above and below the origin line at zero. *p < 0.05.

Analysis items conform to a normal distribution (*p* > 0.05). The Pearson correlation coefficient between the overall app quality, MARS sub-score categories (engagement, functionality, esthetics, and information), and subjective quality (user rating) in China and the United States is shown in Figure 5. Except for the functionality quality score in the United States (rho = 0.37, *p* < 0.05), the user rating was not significantly correlated with the overall MARS score or sub-scores (*p* > 0.05) in either the US or Chinese apps.

## 4. Discussion

This research examined the basic characteristics and functionalities of in-store mobile apps for PtP N&PA in the United States and China, two of the major app markets. The study provides an overview of their key characteristics and functions, focusing on flaws and gaps to be addressed in future eHealth-related innovations. We consider such a survey to be crucial for designing or updating new high-quality maternal N&PA apps during the COVID-19 pandemic. Through apps, prenatal and postnatal education and support offered by a multidisciplinary team of professionals can provide suitable guidance on healthy N&PA throughout the PtP period. In summary, our research demonstrates that (1) there are significant disparities in PtP N&PA apps between the United States and China; (2) the apps from both countries faced the challenges of lacking evidence-based information; and (3) user rating and overall app quality are unrelated. These findings contradict our predictions and point to critical areas for improvement in N&PA apps for pre- and postpartum women.

### 4.1. Hypothesis validation results overview

#### 4.1.1. App-specific metadata

All 31 US apps were divided into three categories: food and drink, health and fitness, and medical (Table 1). Several Chinese apps were education- and life-based, with one remaining uncategorized. A possible explanation for these discrepancies is that the US Food and Drug Administration (FDA) has established regulatory guidelines for classifying mobile health apps into health management, general management, or medical devices categories (39). Unlike in the United States, Chinese regulations governing mobile apps remain in the trial implementation. Additionally, there are over 400 Android app stores in China, making it difficult to standardize categorization criteria. Furthermore, each Chinese app catered to a wider spectrum of consumers than equivalent US apps. As a result, the developers of US apps should customize their products to meet the needs of a wider range of populations. According to recent research, the demands of Chinese pregnant and postpartum women are significantly different from those of US women, especially confinement in childbirth in China, which may account for the disparities among target users (40). Additionally, two Chinese apps evaluated in this research specifically targeted TTC male users. Studies indicated sperm quality in men is significant important for fetal health, and moderate exercise and dietary supplements could effectively increase sperm quality in males TTC (41). The development of N&PA programs for TTC male users is therefore necessary.

An absence of privacy policies and safety statements was more pronounced among Chinese apps than US apps, with nearly half of all assessed Chinese apps lacking a privacy policy or safety statement (Table 1). In recent years, illicit data gathering, data leakage, and online crimes based on user data have severely harmed consumers’ legitimate rights and interests (42). We recommend that consumers refrain from installing programs that display excessive permission-seeking or lack privacy declarations and that government authorities increase internet monitoring. More importantly, more than half of the Chinese and US apps did not include citations or identify their scientific authority for the content they provided. The mean score for information quality was the lowest of all the MARS sub-scores in both China and the United States. Given the benefits of mHealth apps for maternal health and the fact that this population may be more susceptible to misleading information (43, 44), it is critical that they provide accurate, comprehensive, and trustworthy N&PA information throughout the pre- and postpartum periods. This may be a challenge for developers because they may lack relevant knowledge, so a multidisciplinary professional development team may be a requirement to ensure the quality of the app in the future.

Almost three-quarters (71%) of US apps contained in-app purchasing; this may be due to US apps including a plethora of fitness-related pregnancy training and postpartum recovery courses (Table 1). In comparison to pregnancy in China, pregnancy in Western countries tends toward fewer taboos and more physical activity (40, 45), which may explain the significant in-app purchase discrepancy between apps in the two nations. High-quality, convenient online prenatal fitness sessions can benefit maternal and fetal health (46). Including a fitness component in Chinese maternity apps is important, especially considering the current COVID-19 pandemic. Furthermore, there were significant linguistic discrepancies between the US and Chinese apps. An app is available in a virtual store; therefore, the user would likely have to select one in their own language. In addition, having a multi-language system is unnecessary because popular apps used by individuals in other nations sometimes have specialized overseas versions in China.

The quality of an app impacts not only the number of active users but also the capital growth of the firm that created it (35). User feedback (subjective quality) is a way for new users to immediately assess an app’s quality, and research has shown it to be highly influential in convincing new users to download an app (29). The quality of an app comprises user engagement, information, functionality, and esthetics (25). Both countries showed satisfactory levels of subjective and app quality, with China slightly surpassing the United States in both aspects (Figure 4). N&PA app development in the future should be enhanced from an acceptable quality level to one that is convenient, reliable, and high quality that meets the needs of target consumers. The Pearson correlation analysis revealed no association between user rating and app quality (Figure 5); therefore, our hypothesis predicting a link between app quality and user rating was not supported by the above finding. We suggest that user ratings should not be utilized as a stand-alone element to determine the quality of an app and whether to install it as a user.

#### 4.1.2. Functionalities and modules

According to our findings, the most frequently included modules set by developers in prenatal N&PA apps in China and US were monitoring, general education, nutrition, exercise, community, and purchasing (Figure 2).

General education characteristics varied substantially between China and the United States (Figure 3). Pregnancy diet and exercise taboo knowledge were more prevalent in Chinese apps, while nutrition knowledge for women TTC and pregnancy with type 2 diabetes were more prevalent in US apps (Figure 3). This distinction may be due to the cultural differences between Chinese and Western pregnancy, as discussed above (40). In addition, the pregnancy guidelines developed in recent years by the United States and European nations, which include nutrition and exercise recommendations as well as information on prenatal examination, obesity, and other pregnancy complications at all stages of pregnancy may contribute to these differences (47, 48). When pregnant women acquire nutritional knowledge, they are better prepared to avoid common nutritional errors, create healthy eating habits, and manage their weight during pregnancy (49). This implies that app-based general education in the United States and China should include complete information for the entire PtP period, as it would be beneficial to maternal and fetal health.

Compared to Chinese apps, US apps included more exercise-related content (i.e., pregnancy yoga, fitness, and meditation) (Figure 3). The possible reasons for this difference in pre- and postpartum exercise modules have been previously addressed. Despite evidence demonstrating the advantages of exercise during pregnancy, many pregnant women do not engage in the recommended amount of physical activity due to a lack of financial resources or expert support, in addition to isolation during the COVID-19 pandemic (15, 16). Because online courses are more affordable than those provided by professional organizations, we recommend that Chinese apps be improved to address this issue of accessibility by including exercise modules. This would ensure that users can access beneficial exercise modules, even in impoverished areas and during COVID-19-related isolation.

Nutrition modules mainly consisted of recipe suggestions (i.e., three meals a day, dessert, and snacks), cooking methods, nutritional components, and calories in food (Figure 2). In the Chinese apps, recipe guidance that included cooking steps, calories, and nutrition facts was more common than in the US apps. Given that Chinese food involves more intricate processes and ingredients than Western cuisine, detailed guidance may be necessary to match consumer expectations. Meanwhile, neither the Chinese nor the US apps offered any nutrition advice specific to before, during, or after exercise. According to the International Society of Sports Nutrition, proper nutritional intake before, during, and after exercise would significantly enhance exercise capacity and maintenance of normal physical function (50). We propose that app developers highlight the efficient combination of diet and exercise to promote maternal and fetal health.

### 4.2. Strengths and limitations

Several merits and limitations should be addressed in this research before evaluating the conclusions. We contribute to the literature in the following ways. First, apps from two nations with very different healthcare systems, cultures, and economic statuses were compared. This allowed us to evaluate app functionality and usage on a more global scale. Second, this research is the first to examine the link between user rating and app quality associated with in-store PtP N&PA apps in the United States and China. Third, we behaved as users at different PtP periods to gather data and ensure we did not miss critical information. Finally, the collected data were rigorously examined to verify that they were trustworthy and consistent prior to performing the analysis. There were also some limitations, as follows. First, we reviewed the apps at a single point in time, which means we may have ignored modifications to app functionality over the long term. Second, we also removed paid apps without free trials, which could have prevented us from accessing all the available information. Third, the possibility of evaluation bias should be acknowledged. Although app quality was independently assessed by six researchers and agreement was high, it is possible that ratings were subject to individual preference. Finally, a language barrier prevented us from including apps from other countries. Future investigators may seek to examine other time periods to produce longitudinal comparisons of functional modules and quality across a larger number of countries.

## 5. Conclusion

In summary, the basic characteristics and functional modules of in-store mobile PtP N&PA apps varied between the United States and China. Apps from both counties, but notably those from China, shared common deficits, including a lack of evidence-based information, the potential for misleading material, and the absence of empirical app assessments. Chinese apps had a higher concentration of educational content, while US apps had a higher concentration of workout-related information. The app quality of both countries was considered satisfactory, and there was no correlation between app quality and user rating. These results highlight the need to provide the reliable information of PtP N&PA apps present in both nations and suggest that user ratings cannot be used as an objective indicator of app quality. Effective regulation of app information is required to ensure the quality of in-store apps. Women’s basic public health-related services may be promoted *via* the creation of highly effective PtP apps.

## Data availability statement

The raw data supporting the conclusions of this article will be made available by the authors, without undue reservation.

## Author contributions

HY: conceptualization, funding acquisition, software, validation, visualization, and writing of the original draft. HY, JH, KL, WQ, and JL: data curation and formal analysis. HY and JH: methodology. HY and AS: project administration, resources, supervision, and writing of the review and editing. All authors revised and approved the manuscript.
