# Supplementary material for: Quality assessment of pre- and postnatal nutrition and exercise mobile applications in the United States and China
Source: Front Nutr. 2023 Jan 9;9:942331. doi: 10.3389/fnut.2022.942331 (PMC9868741; doi:10.3389/fnut.2022.942331)
Supplement: Supplementary file 1 [file Table_1.DOCX]

**Supplementary Table 1**

**Content analysis of pre- and postnatal nutrition and exercise mobile apps in the United States and China**

**Hongli Yu^1,2*^, Juan He^1^, Keqiang Li^1^, Wen Qi^1^, Jiahui Lin^3^, Anna Szumilewicz^1^**

^1^ Department of Sport, Gdansk University of Physical Education and Sport, Gdansk, Poland

^2^ Jiuling Primary School, Mianyang city, Sichuan province, China

^3^ Football Academy, Beijing Sports University, Beijing, China

*** Correspondence:**Hongli Yu
hongli.yu@awf.gda.pl

**Supplementary Table 1**. Mobile Application Rating Scale scores for the 65 evaluated apps.

|  |  | MARS Score Dimension | | | | |
| --- | --- | --- | --- | --- | --- | --- |
| Apps | Country | Engagement mean score | Functionality mean score | Aesthetics mean score | Information mean score | App quality mean score |
| Yun Fu Yun Qi Shi Pu | China | 2.80 | 3.25 | 2.33 | 2.33 | 2.68 |
| Huai Yun Guan Jia | China | 4.80 | 4.75 | 4.33 | 3.50 | 4.35 |
| Yun Fu Yin Shi Jing Ji | China | 2.80 | 3.00 | 3.67 | 3.17 | 3.16 |
| Yue Zi Shi Pu | China | 2.80 | 3.00 | 4.00 | 3.00 | 3.20 |
| Yun Fu Yun Qi Ying Yang Shi Pu | China | 3.20 | 3.00 | 3.00 | 3.00 | 3.05 |
| Lan Fan | China | 2.80 | 3.00 | 3.33 | 2.33 | 2.87 |
| Mu You Yue Jing Qi Zhu Shou | China | 4.80 | 4.50 | 4.67 | 4.29 | 4.56 |
| Ma Ma Bang | China | 4.80 | 4.50 | 4.67 | 4.17 | 4.53 |
| Yue Xue Yuan | China | 5.00 | 4.50 | 4.67 | 4.33 | 4.63 |
| Ying Er Shi Pu | China | 3.00 | 2.75 | 3.00 | 2.67 | 2.85 |
| Yun Fu Shi Pu 1 | China | 3.00 | 3.00 | 3.33 | 2.67 | 3.00 |
| Yun Qi Ban Lv | China | 5.00 | 4.25 | 5.00 | 4.43 | 4.67 |
| Yun Fu Shi Pu 2 | China | 3.00 | 3.75 | 3.33 | 2.83 | 3.23 |
| Ma Ma Yu Er Fu Shi Da Quan | China | 3.80 | 3.25 | 4.00 | 3.17 | 3.55 |
| Ma Ma She Qu | China | 4.40 | 4.25 | 4.00 | 4.29 | 4.23 |
| Bao Bao Shi Pu | China | 3.20 | 3.50 | 3.33 | 2.67 | 3.18 |
| Bei Yun Shi Pu | China | 3.20 | 3.75 | 3.33 | 2.50 | 3.20 |
| Yun Qi Shi Pu 1 | China | 3.20 | 3.25 | 3.33 | 2.50 | 3.07 |
| Ma Ma Wang Yun Yu-Pro | China | 4.80 | 4.50 | 4.33 | 4.14 | 4.44 |
| Bao Bao Fu Shi 1 | China | 3.00 | 3.75 | 3.33 | 2.83 | 3.23 |
| Hao Yun Ma | China | 4.00 | 4.25 | 4.33 | 3.86 | 4.11 |
| Bao Bao Fu Shi 2 | China | 3.20 | 3.25 | 4.00 | 2.67 | 3.28 |
| Ding Xiang Ma Ma | China | 3.4 | 4.3 | 4.7 | 3.7 | 4.0 |
| Bao Bao Guan Jia | China | 3.20 | 3.75 | 3.33 | 2.67 | 3.24 |
| Ke Xue Zuo Yue Zi | China | 3.20 | 3.50 | 3.33 | 2.83 | 3.22 |
| Yun Qi | China | 2.80 | 3.25 | 3.00 | 2.17 | 2.80 |
| G Dong | China | 3.0 | 3.8 | 2.3 | 4 | 3.2 |
| Wu Ban Mi Ban Yun Jiao Yu | China | 4.60 | 4.25 | 4.67 | 4.29 | 4.45 |
| Yun Qi Dao Shi | China | 4.40 | 4.00 | 4.33 | 3.71 | 4.11 |
| Yi Zheng Jia | China | 4.20 | 4.00 | 4.67 | 3.50 | 4.09 |
| Xin Sheng Er Hu Li Bao Dian | China | 3.00 | 3.00 | 3.67 | 2.33 | 3.00 |
| Zuo Yue Zi Bai Ke | China | 3.00 | 3.00 | 3.00 | 2.50 | 2.88 |
| Yun Qi Shi Pu 2 | China | 3.20 | 3.25 | 3.33 | 2.33 | 3.03 |
| Yun Fu Yu Jia | China | 3.2 | 4.5 | 3.3 | 3.5 | 3.6 |
| Baby2Body | US | 3.00 | 4.25 | 3.67 | 3.00 | 3.48 |
| HiMommy | US | 2.00 | 2.00 | 1.67 | 1.67 | 1.83 |
| iBirth | US | 2.80 | 1.75 | 2.00 | 2.00 | 2.14 |
| Healthy Mummy | US | 2.00 | 2.50 | 2.67 | 2.33 | 2.38 |
| Keleya | US | 1.40 | 2.75 | 1.67 | 2.17 | 2.00 |
| FlavorBaby | US | 3.20 | 2.75 | 4.33 | 3.00 | 3.32 |
| Pregnancy Tracker | US | 3.80 | 3.75 | 4.33 | 3.57 | 3.86 |
| Pregnancy Food Guide | US | 2.80 | 3.75 | 3.00 | 3.00 | 3.14 |
| Pregnancy Diet | US | 3.60 | 4.75 | 3.67 | 3.00 | 3.75 |
| Move Your Bump | US | 3.20 | 4.00 | 4.33 | 4.00 | 3.88 |
| Tone It Up | US | 3.20 | 4.00 | 4.00 | 3.83 | 3.76 |
| Fit Body | US | 3.80 | 4.50 | 3.67 | 3.83 | 3.95 |
| Juna | US | 4.00 | 3.75 | 4.33 | 4.00 | 4.02 |
| Fit-Pregnancy | US | 3.80 | 4.50 | 3.67 | 4.00 | 3.99 |
| Keleya | US | 4.00 | 3.25 | 3.33 | 3.60 | 3.55 |
| Crewe Fitness | US | 3.60 | 4.00 | 3.00 | 4.17 | 3.69 |
| MomEats | US | 2.60 | 3.25 | 3.33 | 3.40 | 3.15 |
| Pregnancy Diet App | US | 1.80 | 3.50 | 2.00 | 2.60 | 2.48 |
| Diastasis Recti Fix | US | 2.8 | 3.5 | 3.0 | 2.2 | 2.8 |
| Pregnancy Workouts- Prenatal | US | 4.2 | 4.5 | 4.7 | 3.3 | 4.2 |
| VITA | US | 4.20 | 4.25 | 5.00 | 4.50 | 4.49 |
| Eat For Two | US | 3.00 | 4.50 | 2.67 | 2.50 | 3.17 |
| Kegel Trainer | US | 3.2 | 3.5 | 3.3 | 1.8 | 2.9 |
| Postnatal Pilates | US | 4.0 | 3.3 | 3.7 | 2.7 | 3.4 |
| Pregnancy cooking | US | 1.60 | 3.75 | 3.00 | 3.00 | 2.84 |
| Fertility Diet Guide | US | 2.00 | 3.00 | 2.33 | 3.20 | 2.63 |
| Pregnancy Yoga & Prenatal | US | 4.0 | 4.8 | 4.0 | 3.7 | 4.2 |
| Studio Bloom | US | 3.8 | 4.3 | 3.3 | 4.2 | 3.9 |
| Care Pregnant Mother | US | 3.4 | 4.0 | 2.3 | 1.8 | 2.8 |
| Birthpedia | US | 4.2 | 4.6 | 4.7 | 2.3 | 3.9 |
| Every Mother | US | 4.2 | 4.3 | 4.3 | 4.9 | 4.4 |
